# Challenges in Oral Hygiene and Oral Health Policy

**DOI:** 10.3389/froh.2020.575428

**Published:** 2020-10-07

**Authors:** Duangporn Duangthip, Chun Hung Chu

**Affiliations:** Faculty of Dentistry, The University of Hong Kong, Hong Kong SAR, China

**Keywords:** dental caries, dental plaque, dental public health, health policy, oral health, oral hygiene, periodontal disease, translational research

## Global Burden of Oral Diseases

Oral health is indivisible from general health and well-being. Oral diseases are prevalent worldwide and significantly burden global economies and people's health, considerably reducing the quality of life of those affected. The Global Burden of Disease study reported that oral conditions affected 3.9 billion people [1]. Dental caries (tooth decay) and periodontal disease are the most prevalent oral diseases globally [2]. Approximately half or more of the world's population suffered from periodontal diseases, and about 11.2% suffered from severe periodontitis [3]. Similarly, dental caries was most prevalent in permanent teeth, affecting around 2.4 billion people, whereas early childhood caries is a silent global epidemic, affecting 621 million children [4], negatively affecting their quality of life and well-being [5]. Due to the changes in demographic profiles, including the aging population, the cumulative burden of oral diseases and conditions has increased significantly. The number of people with untreated oral conditions increased from 2.5 billion in 1990 to 3.5 billion in 2015, with a 64% upsurge in Disability Adjusted Life Years (DALYs) throughout the world [6].

## Oral Hygiene: the Link With Systemic Diseases

It has been documented that oral diseases share similar behavioral risk factors with other non-communicable diseases (NCDs), including excess sugar consumption, unhealthy eating habits, smoking, and excessive alcohol consumption [7]. Poor oral health has been associated with the main NCDs, such as cardiovascular diseases and diabetes mellitus [8]. Improved oral hygiene has been linked to improving surrogate measures of cardiovascular diseases by decreasing the progression of the intima-media layers of the carotid artery [9]. Several systematic reviews have showed that improved oral hygiene in diabetes patients reduced their hemoglobin A1C levels [10]. In addition to the association of oral hygiene with chronic illnesses, poor oral hygiene has been found to have a role in the etiology of oral cancer [11].

In geriatric patients, routine oral hygiene, such as brushing teeth after meals, can decrease the incidence of aspiration pneumonia [12]. In general, older adults are at higher risk for dental infections and associated complex complications. Tooth loss can lead to a reduced ability to chew certain foods, possibly leading to malnutrition in late adulthood. Prolonged oral infections may result in systemic infections, including the infection of endocardial implants and artificial joints. Elders with dementia had significantly poorer oral hygiene and more caries experiences than those without dementia [13]. With the global trend of an aging population, good oral hygiene and regular dental care should be promoted in elderly populations to reduce potentially severe dental infections and associated health complications [14]. In pediatric populations, dental caries in deciduous teeth remains highly prevalent worldwide [15]. Oral diseases are chronic in nature and cumulative over the life course. Childhood is a sensitive phase that affects people's lifelong health, not only general health but also oral health. The socioeconomic statuses under which children grow up have a great and long-lasting influence on their level of oral diseases in adulthood [16].

## Poor Oral Hygiene: Main Etiology of Oral Diseases

Poor oral hygiene is associated not only with systemic diseases, but also with several oral diseases. In children, poor oral hygiene has been found to be the main cause of early childhood caries (ECC) [17]. From birth to toddlerhood, infants and toddlers who had heavy plaque accumulation were at higher risk of developing caries and having severe ECC [18]. Similarly, preschool children who had a significantly higher plaque index score developed more caries than those with lower plaque scores [19]. Nevertheless, the effect of oral hygiene *per se* on caries development is challenging to determine, since many studies have been confounded by fluoridated dentifrices [20]. In the presence of fluorides, oral hygiene procedures are effective for preventing and controlling dental caries when plaque removal is performed properly [21]. However, a recent systematic review and meta-analysis revealed that in the absence of fluorides, the benefit of personal oral hygiene alone in reducing the incidence of dental caries is questionable [22]. Nevertheless, this conclusion should not prevent clinicians from advising their patients on the potential benefits of oral hygiene for caries control. Personal oral hygiene is considered a pleasant, practical, and cost-effective measure to deliver fluoride daily. In contrast to periodontal diseases, plaque accumulation and inadequate personal oral hygiene have been documented as crucial risk factors for periodontitis. Fair to poor oral hygiene escalates the periodontitis risk by 2–5 times [23]. In addition to being a major cause of oral diseases, poor oral hygiene significantly influences the success of minimally invasive interventions [24].

## Oral Health Policy: Challenges and Opportunities

Oral diseases and conditions have persisted as a public health challenge globally, with certain problems in many countries associated with income inequality and commercial changes. Although these oral diseases are mostly preventable, they continue to be highly prevalent, reflecting inequalities and insufficient financial resources, especially in deprived communities or low- and middle-income countries [25]. As with the majority of these diseases, oral diseases and conditions are socially patterned. Children living in poverty and socially marginalized groups are the most affected by dental caries and have poor access to dental care [26]. In developing countries, the vast majority of oral diseases remain untreated because the treatment costs exceed available financial and human resources [25]. These costs impose economic burdens not only on families, but also on health care sectors. Currently, a wider social framework including behavioral, biological, psychosocial, economic, and political determinants is suggested as a better approach to explore the etiology of chronic illnesses, including oral diseases, and patterns of health inequalities [27]. Significant oral health inequalities remain within and between countries. A vicious cycle of unfair economic arrangements, poor health policies, and bad governance is accountable for the majority of health inequalities that the world is facing. The existing neglect in oral health policy and the socioeconomic and commercial determinants should be further addressed. The paradigm shift from a surgical to a medical model has been advocated to pursue the utmost intention of maintaining a disease-free mouth and is anticipated to improve people's oral health-related quality of life [28]. Minimally invasive interventions are effective and should be implemented whenever possible to postpone or reduce the need for extensive restorations and surgical interventions [29]. Although several theoretical concepts and strategies have been published, there are limited studies that encompass the suitability and applicability of these concepts in large clinical trials or a real community setting.

## High Risk vs. Population Approach

Two strategies namely *high risk* and *population* have been proposed and debated [30]. The *high risk* strategy aims to target prevention at the high-risk tail of the distribution by providing preventive measures to susceptible patients. Several preventive actions have been proposed in the literature, e.g., identifying high caries risk children by means of dental screening and providing dental sealant treatments to prevent tooth decay. Studies also investigated how to improve the effectiveness of pit and fissure sealants [31]. Clinical trials and systematic reviews have concluded that the use of topical silver and fluoride applications is effective for preventing and arresting caries [32, 33]. Nevertheless, this approach focuses on the causes of illnesses in each individual and mostly depends on health care providers. Thus, the *high risk* approach alone can be regarded as temporary and palliative since it does not change the societal norms that determine exposure and does not eliminate the underlying causes of why oral diseases continue. In addition, any approach focusing on individual behaviors may also increase oral health inequalities, since those with higher education attainment and greater prosperity—having higher control over their own lives—are likely to respond well to this individualistic approach. The causes of the social distribution of dental illness have not been truly addressed. Despite several decades of recognition of oral health disparities and the policy mandates needed to reduce this problem, low-income, ethnic minority, and rural populations continue to experience inadequate access to quality dental care.

In fact, an individual oral health habit is socially conditioned. Discovering how to change social norms to create healthier lifestyles is a great challenge. Some *population* approaches such as changing oral health-related legislation have been proposed, including sugar taxation [34] with the use of altering incentives for healthy eating habits and water fluoridation [35]. The advantages of this *population* approach are that it is radical and powerful according to the preventive paradox [36]. Ideally, oral health policies should incorporate a universal population approach and target high-risk populations, thus adding significant benefits to the present efforts to advance oral health care equity. Nevertheless, resources are always confined. The inputs, outputs and availability of health care resources should be balanced to ensure their long-term sustainability. Further well-conducted research on this topic is required.

## Challenging Tasks: Closing the Gaps

Several oral health programs or interventions published in the literature have shortcomings, as the fundamental knowledge and translation gaps are not fully acknowledged. Feasible and scalable multilevel interventions, guided by multidisciplinary research collaborations with the engagement of a broad group of stakeholders, are needed, ultimately leading to more sustainable changes than targeting interventions at each individual. Furthermore, collaboration and networking among oral health care providers, researchers, and policymakers to overcome implementation challenges should also be strengthened. We urge dental researchers to systematically evaluate the programs in underserved populations. Natural experiments on oral health care reform and policies to monitor their impacts should be reported. The demonstration of large-scale community projects using effective evidence-based approaches should be documented. Lastly, studies on how to provide sufficient incentives to providers at the individual and organizational levels are important. Research on payment and delivery reform alongside related legislative and regulatory changes is limited. Future work on these issues is required to make oral health care delivery more sustainable, with promising outcomes and affordable treatments costs that are truly beneficial.

## Let's Work Together

A number of dental journals are loaded with laboratory studies which investigate the understanding of various biochemical processes at a micro level. Unfortunately, they often have limited community and clinical relevance, seldom have pathways to benefit the end users, or never demonstrate the impacts beyond academia. In this section, we have called for studies on revisiting oral health policies and the development of effective evidence-based strategies to encourage changes in oral health-related behaviors to prevent the impending epidemic of oral diseases. Studies including but not limited to those on innovative drugs and materials, digital dentistry, intervention programs for unmet needs to help address how to improve oral hygiene and tackle the burden of dental caries, tooth wear, and periodontal and mucosal diseases would be most welcome. In addition, implementation research and community participatory-based research studies, including reports on the impacts of oral health policies, are highly encouraged to be submitted for this section.

## Author Contributions

DD: conceptualization and writing—original draft preparation. CC: conceptualization and critically reviewing and editing. All authors have read and agreed to publish this article.

## Conflict of Interest

The authors declare that the research was conducted in the absence of any commercial or financial relationships that could be construed as a potential conflict of interest.
